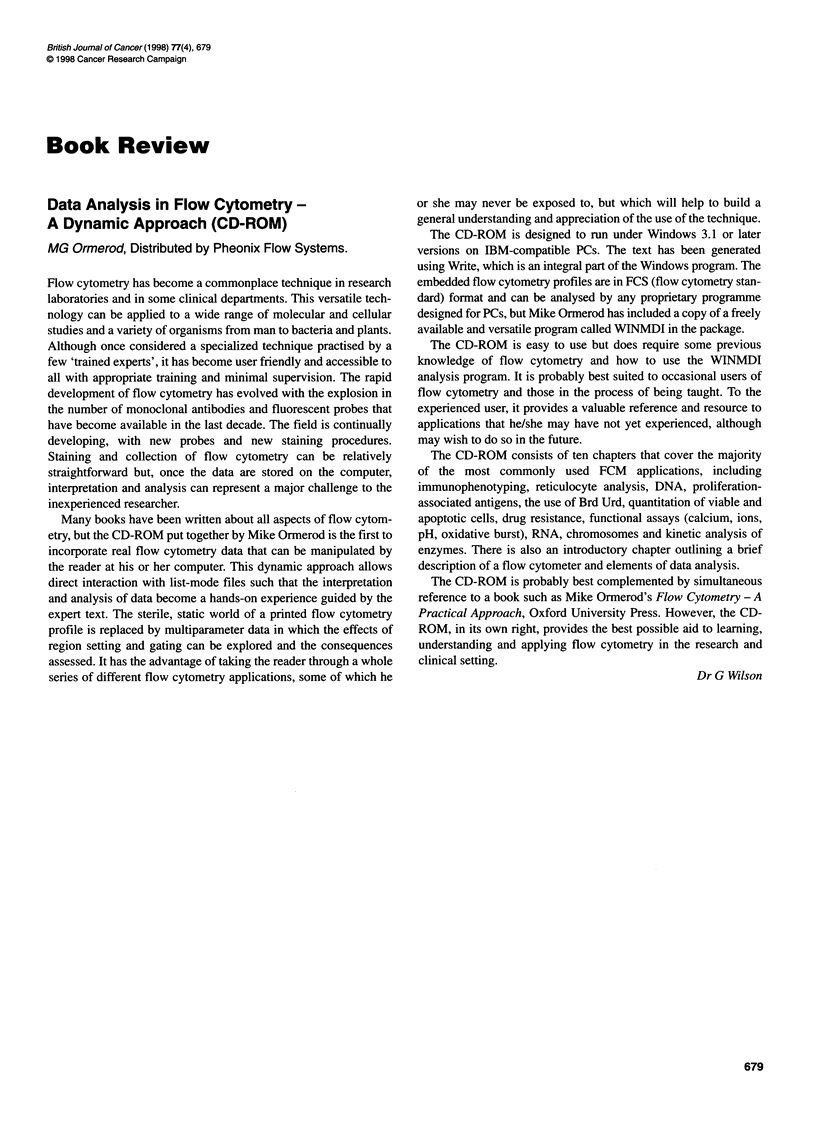# Data Analysis in Flow Cytometry - A Dynamic Approach (CD-ROM)

**Published:** 1998-02

**Authors:** G Wilson


British Journal of Cancer (1998) 77(4), 679
© 1998 Cancer Research Campaign

### Book Review

### Data Analysis in Flow Cytometry – A Dynamic Approach (CD-ROM)

*MG Ormerod*, Distributed by Pheonix Flow Systems.

Flow cytometry has become a commonplace technique in research laboratories and in some clinical departments. This versatile technology can be applied to a wide range of molecular and cellular studies and a variety of organisms from man to bacteria and plants. Although once considered a specialized technique practised by a few 'trained experts', it has become user friendly and accessible to all with appropriate training and minimal supervision. The rapid development of flow cytometry has evolved with the explosion in the number of monoclonal antibodies and fluorescent probes that have become available in the last decade. The field is continually developing, with new probes and new staining procedures. Staining and collection of flow cytometry can be relatively straightforward but, once the data are stored on the computer, interpretation and analysis can represent a major challenge to the inexperienced researcher.

Many books have been written about all aspects of flow cytometry, but the CD-ROM put together by Mike Ormerod is the first to incorporate real flow cytometry data that can be manipulated by the reader at his or her computer. This dynamic approach allows direct interaction with list-mode files such that the interpretation and analysis of data become a hands-on experience guided by the expert text. The sterile, static world of a printed flow cytometry profile is replaced by multiparameter data in which the effects of region setting and gating can be explored and the consequences assessed. It has the advantage of taking the reader through a whole series of different flow cytometry applications, some of which he or she may never be exposed to, but which will help to build a general understanding and appreciation of the use of the technique.

The CD-ROM is designed to run under Windows 3.1 or later versions on IBM-compatible PCs. The text has been generated using Write, which is an integral part of the Windows program. The embedded flow cytometry profiles are in FCS (flow cytometry standard) format and can be analysed by any proprietary programme designed for PCs, but Mike Ormerod has included a copy of a freely available and versatile program called WINMDI in the package.

The CD-ROM is easy to use but does require some previous knowledge of flow cytometry and how to use the WINMDI analysis program. It is probably best suited to occasional users of flow cytometry and those in the process of being taught. To the experienced user, it provides a valuable reference and resource to applications that he/she may have not yet experienced, although may wish to do so in the future.

The CD-ROM consists of ten chapters that cover the majority of the most commonly used FCM applications, including immunophenotyping, reticulocyte analysis, DNA, proliferation-associated antigens, the use of Brd Urd, quantitation of viable and apoptotic cells, drug resistance, functional assays (calcium, ions, pH, oxidative burst), RNA, chromosomes and kinetic analysis of enzymes. There is also an introductory chapter outlining a brief description of a flow cytometer and elements of data analysis.

The CD-ROM is probably best complemented by simultaneous reference to a book such as Mike Ormerod's *Flow Cytometry – A Practical Approach*, Oxford University Press. However, the CD-ROM, in its own right, provides the best possible aid to learning, understanding and applying flow cytometry in the research and clinical setting.

*Dr G Wilson*